# [*N*,*N*′-Bis(2,3,4-trimeth­oxy­benzyl­idene)­ethane-1,2-diamine-κ^2^ *N*,*N*′]dibromido­mercury(II)

**DOI:** 10.1107/S1600536812030577

**Published:** 2012-07-10

**Authors:** Aliakbar Dehno Khalaji, Michal Dušek, Karla Fejfarová

**Affiliations:** a Department of Chemistry, Faculty of Science, Golestan University, Gorgan, Iran; b Institute of Physics, Na Slovance 2, 182 21 Prague 8, Czech Republic

## Abstract

In the title compound, [HgBr_2_(C_22_H_28_N_2_O_6_)], the Hg^II^ ion is bonded to two Br^−^ ions and two N atoms of the chelating Schiff base ligand in a distorted tetra­hedral geometry. The Schiff base ligand adopts an *E*,*E* conformation. The dihedral angle between the planes of the two halves of the central *N*,*N*′-dimethyl­ethylenediamine part of the ligand is 2.3 (11)°. The crystal studied was twinned by pseudomerohedry [twin law (0-10/-100/00-1)]; the contribution of the minor twin component refined to 0.208 (3).

## Related literature

For related structures, see: Marjani *et al.* (2009 ▶); Mahmoudi & Morsali (2008 ▶); Mahmoudi *et al.* (2008 ▶); Khalaji, Fejfarová & Dušek (2011 ▶); Khalaji, Grivani *et al.* (2011 ▶). For properties of Hg^II^ complexes, see: Morsali & Masoomi (2009 ▶). For properties of complexes of symmetric bidentate Schiff base ligands, see: Dolaz *et al.* (2009 ▶, 2010 ▶); Komatsu *et al.* (2007 ▶). For bond-length data, see: Allen *et al.* (1987 ▶).
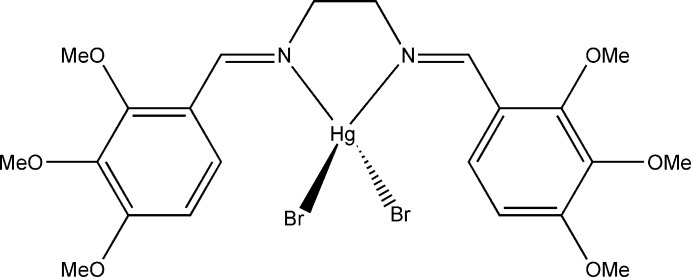


## Experimental

### 

#### Crystal data

[HgBr_2_(C_22_H_28_N_2_O_6_)]
*M* *_r_* = 776.85
Triclinic, 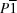

*a* = 7.7847 (1) Å
*b* = 7.7944 (2) Å
*c* = 21.1957 (8) Å
α = 93.487 (2)°
β = 93.163 (2)°
γ = 96.912 (2)°
*V* = 1271.84 (6) Å^3^
*Z* = 2
Mo *K*α radiation
μ = 9.25 mm^−1^
*T* = 150 K
0.23 × 0.16 × 0.06 mm

#### Data collection

Agilent Xcalibur diffractometer with an Atlas (Gemini ultra Cu) detector
Absorption correction: analytical (*CrysAlis PRO*; Agilent, 2011 ▶) *T*_min_ = 0.268, *T*_max_ = 0.694
16635 measured reflections
5193 independent reflections
4391 reflections with *I* > 3σ(*I*)
*R*_int_ = 0.035

#### Refinement

*R*[*F* ^2^ > 3σ(*F* ^2^)] = 0.044
*wR*(*F* ^2^) = 0.125
*S* = 1.76
5193 reflections
299 parameters
H-atom parameters constrained
Δρ_max_ = 1.46 e Å^−3^
Δρ_min_ = −1.48 e Å^−3^

### 

Data collection: *CrysAlis PRO* (Agilent, 2011 ▶); cell refinement: *CrysAlis PRO*; data reduction: *CrysAlis PRO*; program(s) used to solve structure: *SIR2002* (Burla *et al.*, 2003 ▶); program(s) used to refine structure: *JANA2006* (Petříček *et al.*, 2006 ▶); molecular graphics: *DIAMOND* (Brandenburg & Putz, 2005 ▶); software used to prepare material for publication: *JANA2006*.

## Supplementary Material

Crystal structure: contains datablock(s) global, I. DOI: 10.1107/S1600536812030577/wm2653sup1.cif

Structure factors: contains datablock(s) I. DOI: 10.1107/S1600536812030577/wm2653Isup2.hkl

Additional supplementary materials:  crystallographic information; 3D view; checkCIF report

## Figures and Tables

**Table 1:** Selected bond lengths (Å)

Hg1—Br1	2.4798 (13)
Hg1—Br2	2.4832 (14)
Hg1—N1	2.411 (9)
Hg1—N2	2.385 (8)

**Fig. 1.**
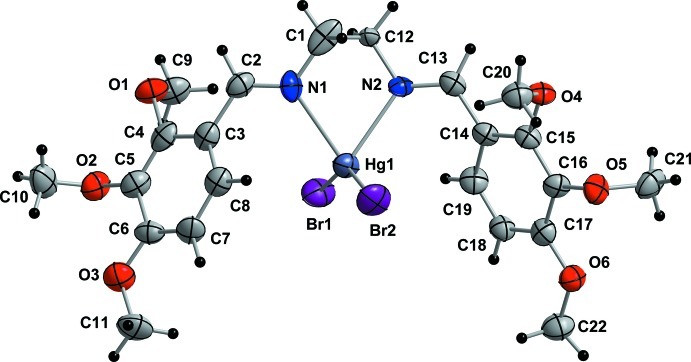
Molecular structure of the title compound. Displacement ellipsoids are drawn at the 50% probability level.

## supplementary crystallographic information

### Comment

Complexes of symmetric bidentate Schiff base ligands with transition metals have attracted much attention because of their catalytic (Komatsu *et al.*, 2007) and antibacterial activity, electrochemical and photophysical properties (Dolaz *et al.*, 2009, 2010). The coordination behavior of Schiff base ligands depends on the metal ion, the reaction condition and the nature of anion and the solvent used. There is a substantial interest in the coordination chemistry of the Hg(II) ion (Marjani *et al.*, 2009; Mahmoudi & Morsali, 2008; Mahmoudi *et al.*, 2008; Khalaji, Fejfarová & Dušek, 2011; Khalaji, Grivani, Rezaei *et al.*, 2011; Morsali & Masoomi, 2009), because of its toxic environmental effects. *N*,*N*' The molecular structure of the title compound, [HgBr_2_(C_22_H_28_N_2_O_6_)], (I), with the atom-numbering scheme is presented in Fig. 1. Bond lengths and angles (Allen *et al.*, 1987) are generally normal. The Hg(II) ion is coordinated by the bidentate Schiff-base ligand (2,3,4-MeO-ba)_2_en and two Br^-^ ions. Although a tetrahedral geometry might be expected for a four coordinated Hg(II) ion, the geometry around Hg(II) is distorted by the restricting bite angle N1—Hg1—N2 (72.1 (3)°) of the chelating Schiff-base ligand. On the contrary, the Br1—Hg1—Br2 angle has opened up to 144.61 (5)°. The N—Hg—Br angles are also distorted from the tetrahedral values. The dihedral angles between the planes defined by atoms C1—N2—C2 and C3–C8, and C12—N2—C13 and C14–C19 are 39.2 (10)° and 40.5 (10)°, respectively. The torsion angles C4—C5—O2—C10 and C15—C16—O5—C21 are -98.9 (13)° and -100.6 (11)°, respectively.

The average Hg—N bond length of 2.40 Å agrees well with the corresponding distances in other tetrahedral Hg(II) complexes (Marjani *et al.*, 2009; Mahmoudi & Morsali, 2008; Mahmoudi *et al.*, 2008; Khalaji, Fejfarová & Dušek, 2011; Khalaji, Grivani, Rezaei *et al.*, 2011; Morsali & Masoomi, 2009). The Schiff-base ligand (2,3,4-MeO-ba)_2_en adopts an *E,E* conformation in this complex.

### Experimental

To a stirring solution of the (2,3,4-MeO-ba)_2_en ligand (0.2 mmol, in 5 ml of chloroform) was added HgBr_2_ (0.2 mmol) in 10 ml of methanol and the mixture was stirred for 10 min in air at room temperature and was then left at 273 K for several days without disturbance yielding suitable crystals of (I) that subsequently were filtered off and washed with Et_2_O. Yield: 72%. Colourless crystals. *Anal*. Calc. for C_22_H_28_N_2_O_6_HgBr_2_: C, 34.01; H, 3.63; N, 3.61%. Found: C, 34.15; H, 3.71; N, 3.68%. ^1^H-NMR (CDCl_3_, *δ*(p.p.m.)): 3.73 (s, 6H), 3.77 (s, 6H), 3.82 (s, 6H), 3.86 (s, 4H), 6.87 (d, 2H), 7.63 (d, 2H), 8.58 (s, 2H).

### Refinement

The hydrogen atoms were added geometrically, with a C–H distance of 0.96 Å, and refined as riding on their parent atoms. The methyl H atoms were allowed to rotate freely about the adjacent C—C bonds. The thermal displacement coefficients *U*_iso_(H) were set to 1.5*U*_eq_(C) for the methyl groups and to to 1.2*U*_eq_(C) for the CH– and CH_2_-groups.

The structure of (I) can also be refined in space group *C*2/*c* with unit cell parameters of *a* = 10.332 Å, *b* =11.6601 Å, *c* = 21.1957 Å, *β*=95.017 ° to a relatively good *R* value of 0.055. In the monoclinic structure model two halves of the structure are symmetry-equivalent as found in the ^1^H-NMR solution spectra. However, the true crystal symmetry is triclinic due to small rotations of aromatic rings as well as methyl groups.

The lowering of symmetry can be indicated by comparison of *R*_int_ factors which are 0.035 for triclinic symmetry but almost 0.1 for monoclinic symmetry. In order to test that the triclinic structure model does not contain hidden monoclinic symmetry we used a simulated data set based on the refined triclinic structure, transformed to the twofold monoclinic unit cell and merged according to the monoclinic Laue group. The obtained *R*_int_ of 0.1 was in agreement with the value found experimentally and confirmed the fact that tiny rotations of methyl groups and aromatic rings are responsible for lowering of symmetry from monoclinic to triclinic. Twofold rotation along *b* was used as the twinning operation, which became (0 1 0 / 1 0 0 / 0 0 1) after transformation to the final triclinic unit cell (the matrix acts to indices as columns). The refined twin ratio of the second twin domain was 0.208 (3).

The highest residual electron density of 1.46 e Å^-3^ was located 1.814 (13) Å from C9; the deepest hole of -1.48 e Å^-3^ was located 2.187 (13) Å from C9.

### Crystal data

**Table:**

[HgBr_2_(C_22_H_28_N_2_O_6_)]	*Z* = 2
*M**_r_* = 776.85	*F*(000) = 744
Triclinic, *P*1	*D*_x_ = 2.028 Mg m^−^^3^
Hall symbol: -P 1	Mo *K*α radiation, λ = 0.7107 Å
*a* = 7.7847 (1) Å	Cell parameters from 8498 reflections
*b* = 7.7944 (2) Å	θ = 2.9–26.3°
*c* = 21.1957 (8) Å	µ = 9.25 mm^−^^1^
α = 93.487 (2)°	*T* = 150 K
β = 93.163 (2)°	Plate, colourless
γ = 96.912 (2)°	0.23 × 0.16 × 0.06 mm
*V* = 1271.84 (6) Å^3^	

### Data collection

**Table:**

Agilent Xcalibur diffractometer with an Atlas (Gemini ultra Cu) detector	5193 independent reflections
Radiation source: X-ray tube	4391 reflections with *I* > 3σ(*I*)
Graphite monochromator	*R*_int_ = 0.035
Detector resolution: 10.3784 pixels mm^-1^	θ_max_ = 26.4°, θ_min_ = 2.9°
Rotation method data acquisition using ω scans	*h* = −9→9
Absorption correction: analytical (*CrysAlis PRO*; Agilent, 2011)	*k* = −9→9
*T*_min_ = 0.268, *T*_max_ = 0.694	*l* = −26→26
16635 measured reflections	

### Refinement

**Table:**

Refinement on *F*^2^	112 constraints
*R*[*F*^2^ > 2σ(*F*^2^)] = 0.044	H-atom parameters constrained
*wR*(*F*^2^) = 0.125	Weighting scheme based on measured s.u.'s *w* = 1/(σ^2^(*I*) + 0.0016*I*^2^)
*S* = 1.76	(Δ/σ)_max_ = 0.007
5193 reflections	Δρ_max_ = 1.46 e Å^−^^3^
299 parameters	Δρ_min_ = −1.48 e Å^−^^3^
0 restraints	

### Special details

**Table:**

Refinement. The refinement was carried out against all reflections. The conventional *R*-factor is always based on *F*. The goodness of fit as well as the weighted *R*-factor are based on *F* and *F*^2^ for refinement carried out on *F* and *F*^2^, respectively. The threshold expression is used only for calculating *R*-factors *etc*. and it is not relevant to the choice of reflections for refinement.The program used for refinement, Jana2006, uses the weighting scheme based on the experimental expectations, see _refine_ls_weighting_details, that does not force *S* to be one. Therefore the values of *S* are usually larger than the ones from the *SHELX* program.

### Fractional atomic coordinates and isotropic or equivalent isotropic displacement parameters (Å^2^)

**Table:**

	*x*	*y*	*z*	*U*_iso_*/*U*_eq_	
Hg1	0.37072 (5)	0.63869 (6)	0.25091 (3)	0.04023 (14)	
Br1	0.51422 (17)	0.90658 (15)	0.20946 (8)	0.0588 (5)	
Br2	0.09847 (15)	0.50076 (19)	0.29179 (8)	0.0632 (5)	
O1	0.9444 (9)	0.9090 (11)	0.3853 (4)	0.049 (3)	
O2	0.8619 (10)	1.2308 (10)	0.4242 (4)	0.049 (3)	
O3	0.5366 (11)	1.2813 (12)	0.4428 (4)	0.056 (3)	
O4	0.1026 (9)	0.0570 (9)	0.1171 (4)	0.038 (2)	
O5	−0.2179 (10)	0.1352 (9)	0.0797 (4)	0.046 (3)	
O6	−0.2707 (9)	0.4636 (10)	0.0597 (4)	0.044 (3)	
N1	0.5962 (12)	0.5309 (11)	0.3140 (4)	0.042 (3)	
N2	0.4779 (10)	0.4141 (10)	0.1887 (4)	0.031 (3)	
C1	0.6352 (14)	0.3621 (17)	0.2877 (6)	0.052 (4)	
C2	0.6924 (14)	0.6232 (15)	0.3559 (6)	0.045 (4)	
C3	0.6465 (14)	0.7939 (15)	0.3827 (5)	0.043 (4)	
C4	0.7755 (15)	0.9297 (16)	0.3960 (5)	0.044 (4)	
C5	0.7351 (14)	1.0908 (16)	0.4156 (6)	0.046 (4)	
C6	0.5619 (15)	1.1177 (15)	0.4235 (6)	0.042 (4)	
C7	0.4355 (14)	0.9766 (16)	0.4137 (6)	0.049 (4)	
C8	0.4796 (14)	0.8155 (16)	0.3942 (5)	0.045 (4)	
C9	0.9982 (15)	0.9674 (17)	0.3270 (6)	0.054 (5)	
C10	0.9221 (18)	1.2691 (18)	0.4866 (7)	0.067 (5)	
C11	0.3609 (16)	1.3144 (18)	0.4472 (7)	0.063 (5)	
C12	0.6384 (12)	0.3677 (13)	0.2157 (5)	0.037 (3)	
C13	0.3914 (13)	0.3135 (13)	0.1451 (5)	0.038 (4)	
C14	0.2226 (13)	0.3556 (13)	0.1190 (5)	0.034 (3)	
C15	0.0832 (13)	0.2238 (12)	0.1074 (5)	0.034 (3)	
C16	−0.0803 (13)	0.2636 (13)	0.0872 (5)	0.037 (3)	
C17	−0.1055 (14)	0.4365 (14)	0.0773 (5)	0.039 (4)	
C18	0.0351 (14)	0.5662 (14)	0.0867 (5)	0.040 (4)	
C19	0.1952 (15)	0.5265 (13)	0.1071 (5)	0.041 (4)	
C20	0.0482 (16)	0.0059 (16)	0.1769 (6)	0.050 (4)	
C21	−0.2585 (15)	0.0723 (17)	0.0148 (7)	0.057 (5)	
C22	−0.3020 (16)	0.6398 (15)	0.0520 (7)	0.055 (5)	
H1a	0.546622	0.273063	0.297768	0.0627*	
H1b	0.746606	0.340413	0.304904	0.0627*	
H2	0.798064	0.583586	0.371247	0.0537*	
H7	0.316804	0.990139	0.420474	0.0587*	
H8	0.391237	0.717756	0.38861	0.0538*	
H9a	1.120159	0.961369	0.324628	0.0804*	
H9b	0.935506	0.895556	0.292816	0.0804*	
H9c	0.97558	1.085057	0.32375	0.0804*	
H10a	1.022703	1.353925	0.488537	0.1007*	
H10b	0.833294	1.314124	0.509965	0.1007*	
H10c	0.952002	1.16573	0.504597	0.1007*	
H11a	0.357791	1.43634	0.455575	0.095*	
H11b	0.296018	1.275971	0.408037	0.095*	
H11c	0.310931	1.252928	0.481018	0.095*	
H12a	0.732679	0.450538	0.205595	0.0442*	
H12b	0.657586	0.256372	0.197498	0.0442*	
H13	0.436087	0.210774	0.129318	0.0457*	
H18	0.019991	0.683646	0.078828	0.048*	
H19	0.29061	0.617314	0.113552	0.0489*	
H20a	0.090503	−0.101462	0.18577	0.0748*	
H20b	0.093651	0.093536	0.209341	0.0748*	
H20c	−0.076121	−0.00902	0.176057	0.0748*	
H21a	−0.340801	−0.030218	0.013085	0.0848*	
H21b	−0.307063	0.159617	−0.007986	0.0848*	
H21c	−0.154633	0.04556	−0.003968	0.0848*	
H22a	−0.419337	0.64121	0.035716	0.0829*	
H22b	−0.283965	0.704837	0.092307	0.0829*	
H22c	−0.223713	0.690913	0.023017	0.0829*	

### Atomic displacement parameters (Å^2^)

**Table:**

	*U*^11^	*U*^22^	*U*^33^	*U*^12^	*U*^13^	*U*^23^
Hg1	0.0312 (2)	0.0343 (2)	0.0545 (2)	−0.00009 (12)	0.00690 (19)	0.00200 (19)
Br1	0.0540 (7)	0.0337 (6)	0.0879 (10)	−0.0025 (5)	0.0015 (7)	0.0172 (6)
Br2	0.0325 (6)	0.0683 (8)	0.0868 (11)	−0.0070 (6)	0.0170 (6)	0.0017 (8)
O1	0.030 (4)	0.059 (5)	0.061 (6)	0.006 (3)	0.014 (4)	0.014 (4)
O2	0.041 (4)	0.047 (5)	0.060 (6)	−0.001 (4)	0.005 (4)	0.010 (4)
O3	0.049 (5)	0.062 (6)	0.057 (6)	0.005 (4)	0.013 (4)	0.004 (4)
O4	0.038 (4)	0.031 (4)	0.047 (5)	0.005 (3)	0.008 (3)	0.011 (3)
O5	0.045 (4)	0.039 (4)	0.051 (5)	−0.007 (3)	0.000 (4)	0.005 (4)
O6	0.040 (4)	0.040 (4)	0.051 (5)	0.003 (3)	0.001 (4)	0.006 (4)
N1	0.045 (5)	0.036 (5)	0.041 (6)	−0.012 (4)	0.001 (4)	0.001 (4)
N2	0.029 (4)	0.021 (4)	0.044 (5)	0.002 (3)	0.003 (4)	0.007 (4)
C1	0.032 (6)	0.074 (9)	0.051 (8)	−0.015 (5)	0.005 (5)	0.043 (6)
C2	0.032 (6)	0.047 (6)	0.055 (8)	0.000 (5)	0.001 (5)	0.010 (6)
C3	0.037 (6)	0.054 (7)	0.036 (6)	0.001 (5)	0.003 (5)	0.006 (5)
C4	0.042 (6)	0.062 (8)	0.031 (6)	0.003 (6)	0.000 (5)	0.018 (5)
C5	0.038 (6)	0.052 (7)	0.050 (7)	0.003 (5)	0.007 (5)	0.013 (6)
C6	0.042 (6)	0.044 (6)	0.045 (7)	0.012 (5)	0.011 (5)	0.008 (5)
C7	0.033 (6)	0.067 (8)	0.047 (7)	0.007 (6)	0.013 (5)	−0.004 (6)
C8	0.040 (6)	0.055 (7)	0.039 (7)	0.001 (5)	0.005 (5)	0.008 (6)
C9	0.037 (6)	0.065 (8)	0.061 (8)	0.005 (6)	0.014 (6)	0.014 (7)
C10	0.045 (7)	0.064 (9)	0.085 (11)	−0.009 (6)	0.005 (7)	−0.021 (8)
C11	0.052 (8)	0.069 (9)	0.074 (10)	0.020 (7)	0.022 (7)	0.000 (8)
C12	0.023 (5)	0.037 (6)	0.050 (7)	0.010 (4)	−0.003 (4)	−0.015 (5)
C13	0.041 (6)	0.032 (5)	0.042 (7)	0.003 (5)	0.009 (5)	0.004 (5)
C14	0.035 (5)	0.031 (5)	0.038 (6)	0.003 (4)	0.011 (5)	0.003 (5)
C15	0.041 (6)	0.026 (5)	0.035 (6)	0.002 (4)	0.008 (5)	0.009 (4)
C16	0.030 (5)	0.035 (6)	0.045 (7)	0.001 (4)	0.010 (5)	0.005 (5)
C17	0.045 (6)	0.041 (6)	0.033 (6)	0.004 (5)	0.006 (5)	0.007 (5)
C18	0.048 (6)	0.032 (6)	0.040 (6)	0.003 (5)	0.002 (5)	0.005 (5)
C19	0.050 (7)	0.031 (5)	0.042 (7)	0.000 (5)	0.005 (5)	0.015 (5)
C20	0.050 (7)	0.046 (7)	0.055 (8)	0.007 (5)	0.006 (6)	0.012 (6)
C21	0.039 (7)	0.054 (7)	0.074 (9)	−0.004 (5)	−0.012 (6)	0.013 (7)
C22	0.057 (8)	0.050 (7)	0.061 (9)	0.012 (6)	0.003 (7)	0.015 (6)

### Geometric parameters (Å, º)

**Table:**

Hg1—Br1	2.4798 (13)	C8—H8	0.96
Hg1—Br2	2.4832 (14)	C9—H9a	0.96
Hg1—N1	2.411 (9)	C9—H9b	0.96
Hg1—N2	2.385 (8)	C9—H9c	0.96
O1—C4	1.373 (14)	C10—H10a	0.96
O1—C9	1.412 (16)	C10—H10b	0.96
O2—C5	1.376 (13)	C10—H10c	0.96
O2—C10	1.381 (17)	C11—H11a	0.96
O3—C6	1.356 (15)	C11—H11b	0.96
O3—C11	1.429 (16)	C11—H11c	0.96
O4—C15	1.353 (12)	C12—H12a	0.96
O4—C20	1.423 (15)	C12—H12b	0.96
O5—C16	1.370 (12)	C13—C14	1.478 (15)
O5—C21	1.436 (16)	C13—H13	0.96
O6—C17	1.364 (14)	C14—C15	1.402 (13)
O6—C22	1.441 (15)	C14—C19	1.409 (15)
N1—C1	1.469 (16)	C15—C16	1.397 (15)
N1—C2	1.260 (14)	C16—C17	1.412 (16)
N2—C12	1.440 (13)	C17—C18	1.395 (14)
N2—C13	1.281 (13)	C18—C19	1.373 (16)
C1—C12	1.531 (16)	C18—H18	0.96
C1—H1a	0.96	C19—H19	0.96
C1—H1b	0.96	C20—H20a	0.96
C2—C3	1.505 (17)	C20—H20b	0.96
C2—H2	0.96	C20—H20c	0.96
C3—C4	1.373 (15)	C21—H21a	0.96
C3—C8	1.363 (16)	C21—H21b	0.96
C4—C5	1.377 (18)	C21—H21c	0.96
C5—C6	1.407 (16)	C22—H22a	0.96
C6—C7	1.382 (16)	C22—H22b	0.96
C7—C8	1.386 (18)	C22—H22c	0.96
C7—H7	0.96		
			
Br1—Hg1—Br2	144.61 (5)	H10a—C10—H10c	109.4711
Br1—Hg1—N2	103.1 (2)	H10b—C10—H10c	109.4709
Br2—Hg1—N2	105.22 (18)	O3—C11—H11a	109.4716
N1—Hg1—N2	72.1 (3)	O3—C11—H11b	109.4711
Br1—Hg1—N1	104.5 (2)	O3—C11—H11c	109.4716
Br2—Hg1—N1	103.9 (2)	H11a—C11—H11b	109.4715
C4—O1—C9	113.8 (9)	H11a—C11—H11c	109.4707
C5—O2—C10	113.7 (10)	H11b—C11—H11c	109.4708
C6—O3—C11	116.7 (9)	N2—C12—C1	111.1 (9)
C15—O4—C20	113.1 (9)	N2—C12—H12a	109.4708
C16—O5—C21	113.1 (9)	N2—C12—H12b	109.4712
C17—O6—C22	117.3 (8)	C1—C12—H12a	109.4717
C1—N1—C2	123.4 (10)	C1—C12—H12b	109.4711
Hg1—N2—C12	112.5 (6)	H12a—C12—H12b	107.7784
Hg1—N2—C13	126.1 (7)	N2—C13—C14	120.0 (9)
C12—N2—C13	119.5 (9)	N2—C13—H13	119.9921
N1—C1—C12	108.4 (10)	C14—C13—H13	119.9903
N1—C1—H1a	109.4717	C13—C14—C15	119.6 (9)
N1—C1—H1b	109.4718	C13—C14—C19	121.9 (9)
C12—C1—H1a	109.4703	C15—C14—C19	118.5 (9)
C12—C1—H1b	109.4705	O4—C15—C14	121.1 (9)
H1a—C1—H1b	110.5361	O4—C15—C16	118.7 (8)
N1—C2—C3	121.9 (10)	C14—C15—C16	120.2 (9)
N1—C2—H2	119.0299	O5—C16—C15	119.7 (9)
C3—C2—H2	119.0308	O5—C16—C17	120.1 (9)
C2—C3—C4	119.2 (10)	C15—C16—C17	120.2 (9)
C2—C3—C8	121.2 (10)	O6—C17—C16	116.0 (9)
C4—C3—C8	119.6 (11)	O6—C17—C18	124.7 (10)
O1—C4—C3	120.2 (11)	C16—C17—C18	119.3 (10)
O1—C4—C5	119.4 (10)	C17—C18—C19	120.2 (10)
C3—C4—C5	120.2 (11)	C17—C18—H18	119.8968
O2—C5—C4	120.7 (10)	C19—C18—H18	119.8967
O2—C5—C6	118.6 (10)	C14—C19—C18	121.5 (9)
C4—C5—C6	120.6 (10)	C14—C19—H19	119.2318
O3—C6—C5	115.7 (9)	C18—C19—H19	119.2308
O3—C6—C7	126.2 (11)	O4—C20—H20a	109.4713
C5—C6—C7	118.1 (11)	O4—C20—H20b	109.4709
C6—C7—C8	120.2 (11)	O4—C20—H20c	109.471
C6—C7—H7	119.8945	H20a—C20—H20b	109.4717
C8—C7—H7	119.8948	H20a—C20—H20c	109.4707
C3—C8—C7	121.0 (10)	H20b—C20—H20c	109.4716
C3—C8—H8	119.5052	O5—C21—H21a	109.4712
C7—C8—H8	119.5057	O5—C21—H21b	109.4712
O1—C9—H9a	109.4711	O5—C21—H21c	109.4711
O1—C9—H9b	109.4712	H21a—C21—H21b	109.4711
O1—C9—H9c	109.4705	H21a—C21—H21c	109.4714
H9a—C9—H9b	109.4714	H21b—C21—H21c	109.4714
H9a—C9—H9c	109.4712	O6—C22—H22a	109.4711
H9b—C9—H9c	109.4718	O6—C22—H22b	109.4706
O2—C10—H10a	109.4714	O6—C22—H22c	109.4709
O2—C10—H10b	109.4716	H22a—C22—H22b	109.4716
O2—C10—H10c	109.4711	H22a—C22—H22c	109.4722
H10a—C10—H10b	109.4713	H22b—C22—H22c	109.471
			
C3—C4—O1—C9	97.1 (13)	C20—O4—C15—C16	−81.2 (12)
C14—C15—O4—C20	97.1 (12)	C21—O5—C16—C15	−100.6 (11)
C4—C5—O2—C10	−98.9 (14)	C21—O5—C16—C17	82.3 (12)
C15—C16—O5—C21	−100.6 (12)	C22—O6—C17—C16	177.3 (10)
C5—C6—O3—C11	176.1 (11)	C22—O6—C17—C18	−1.7 (16)
C16—C17—O6—C22	177.3 (10)	N1—C2—C3—C4	−141.7 (11)
C9—O1—C4—C3	97.0 (12)	N1—C2—C3—C8	38.6 (17)
C9—O1—C4—C5	−78.2 (13)	C2—C3—C4—C5	174.7 (11)
C10—O2—C5—C4	−98.9 (13)	C2—C3—C8—C7	−174.4 (11)
C10—O2—C5—C6	84.9 (14)	N2—C13—C14—C15	−137.8 (11)
C11—O3—C6—C5	176.1 (11)	N2—C13—C14—C19	40.2 (15)
C11—O3—C6—C7	−6.8 (18)	C13—C14—C15—C16	175.3 (10)
C20—O4—C15—C14	97.1 (12)	C13—C14—C19—C18	−175.8 (10)
